# Influence of Rice Seeding Rate on Efficacies of Neonicotinoid and Anthranilic Diamide Seed Treatments against Rice Water Weevil

**DOI:** 10.3390/insects5040961

**Published:** 2014-12-01

**Authors:** Jason Hamm, Srinivas Lanka, Michael Stout

**Affiliations:** 1 DuPont Crop Protection, Stine-Haskell Research Center, Newark, DE 19711, USA; E-Mail: jason.c.hamm@usa.dupont.com; 2 Louisiana State University Agricultural Center, Department of Entomology, 404 Life Sciences Building, Baton Rouge, LA 70803, USA; E-Mail: slanka@agcenter.lsu.edu

**Keywords:** rice water weevil, seed treatments, seeding rates, neonicotinoids, anthranilic diamides

## Abstract

Rice in the U.S. is frequently seeded at low rates and treated before sowing with neonicotinoid or anthranilic diamide insecticides to target the rice water weevil. A previous study of the influence of seeding rate on rice water weevil densities showed an inverse relationship between seeding rates and immature weevil densities. This study investigated interactive effects of seeding rate and seed treatment on weevil densities and rice yields; in particular, experiments were designed to determine whether seed treatments were less effective at low seeding rates. Four experiments were conducted over three years by varying seeding rates of rice treated at constant per seed rates of insecticide. Larval suppression by chlorantraniliprole was superior to thiamethoxam or clothianidin, and infestations at low seeding rates were up to 47% higher than at high seeding rates. Little evidence was found for the hypothesis that seed treatments are less effective at low seeding rates; in only one of four experiments was the reduction in weevil densities by thiamethoxam greater at high than at low seeding rates. However, suppression of larvae by neonicotinoid seed treatments in plots seeded at low rates was generally poor, and caution must be exercised when using the neonicotioids at low seeding rates.

## 1. Introduction

The rice water weevil, *Lissorhoptrus oryzophilus* Kuschel, is the most destructive early season insect pest of rice, *Oryza sativa* L., in the United States [1]. This pest has recently invaded other rice-producing regions of Asia and Europe and now poses a global threat to rice production [2]. Rice water weevils overwinter as adults in plant debris, leaf litter, and bunch grasses in and around rice fields and riparian habitats [3]. In early spring, after rice is planted, weevils fly to rice fields and begin feeding by scraping the upper epidermis of rice leaves resulting in feeding scars parallel to the veins of leaves [4]. This type of foliar injury probably does not cause economic losses except under unusually heavy weevil infestations. Female weevils oviposit primarily in leaf sheaths of flooded rice plants beneath the water surface [5]. After hatching, neonates mine through the leaf sheaths or shoots and quickly move to the soil, where they feed on or in the roots of flooded plants, completing four instars and a pupal stage in approximately four weeks [6]. Thus, all life stages of the rice water weevil are completed in association with rice plants—adults, eggs, and first instars on above-ground portions of plants, and larvae and pupae on below-ground portions of plants. Severe root pruning by larvae can lead to reduced tillering and lodging of plants at the vegetative stage of growth and reduced panicle densities and grain weights at maturity [7]. Small-plot research and sampling of commercial fields have shown yield losses can exceed 10% in sites where no control measures are adopted [8]). Although populations of weevils are multivoltine, only a single peak of larval abundance causing yield losses is observed in rice fields [3].

The use of insecticidal seed treatments is currently the most widely used tactic for weevil management. In a recent survey of grower’s practices in Louisiana, for example, over 80% of growers and consultants reported using seed treatments in one or more of the fields for which they were responsible [9]. Three seed treatments are registered for rice water weevil management in the U.S.: Dermacor X-100 (DuPont Crop Protection, Wilmington, DE, USA), the active ingredient (AI) of which is the anthranilic diamide insecticide chlorantraniliprole, and two neonicotinoid seed treatments, CruiserMaxx (AI: thiamethoxam; Syngenta Corporation, Greensboro, NC, USA) and NipsitInside (AI: clothianidin; Valent USA, Walnut Creek, CA, USA). All three seed treatments are systemic, and residues of active ingredients are present in roots, shoots and leaves of treated rice plants [10]. Residues of the different active ingredients are, however, distributed differently in plants and these seed treatments differentially affect *L. oryzophilus* life stages [10]. Plants treated as seeds with chlorantraniliprole show no lethality against adults but suppress egg-laying and show high potency against larvae. In contrast, thiamethoxam seed treatment reduces adult survival and egg-laying, but activity against larvae is lower [11,12]. The high below-ground activity of chlorantraniliprole is consistent with the tendency of this chemical to accumulate in root tissues, whereas the high above-ground activity of thiamethoxam is consistent with high concentrations of the active ingredient in above-ground portions of rice [10]. Both insecticides also differ in their persistence, with chlorantraniliprole far more persistent than thiamethoxam in plant tissues.

Rice water weevil populations are also influenced by agronomic practices. Economically damaging infestations can be avoided by planting rice earlier in the growing season [13,14,15]. Water management practices—draining fields after larval infestation, delaying onset of flood, or reducing flood depth—can exploit the dependence of rice water weevils on flooded conditions [5,16,17,18,19]. A third agronomic practice that can influence rice water weevil populations is rice-seeding rate. Several past workers have found high weevil densities in areas of fields with low plant densities [20,21]. More recently, an inverse relationship between seeding rate and densities of rice water weevil larvae and pupae was confirmed by Stout *et al.* [22]; furthermore, these authors found that rice seeded at lower rates (<50 kg/ha) sometimes suffered higher proportionate yield losses from rice water weevils, probably because root-feeding by larval rice water weevils results in reductions in tillering and panicle formation, the processes particularly important to rice yield at low seeding rates [7].

There has been a trend in recent years toward the use of lower seeding rates in U.S. rice [23], a trend attributable primarily to increased adoption of expensive hybrid and herbicide-tolerant seed (25–60 kg of seed per ha). This trend, coupled with the widespread use of seed treatment insecticides, makes it important to investigate possible interactions among seeding rate and seed treatment insecticides. If insecticidal seed treatments affect rice water weevils exclusively via ingestion by adults or larvae of active ingredient that has moved systemically from the seed to other parts of the plant, then the effectiveness of seed treatments should depend only on the per seed treatment rate and not on seeding rate. If, on the other hand, rice water weevil larvae are also affected by contact with insecticide active ingredient that leaches from the seed into the soil surrounding roots, then the effectiveness of seed treatments may be affected by seeding rate. This is because, given a constant per seed treatment rate, a reduction in seeding rate results in less insecticide per unit volume of soil. Alternatively, if reduced plant densities in fields planted at low seeding rates result in increased tillering and greater vegetative growth of remaining plants, insecticide active ingredient may be diluted to a greater extent in plants seeded at a low rate, and effectiveness of seed treatments thereby reduced. As neonicotinoid and anthranilic diamide seed treatments differ with respect to patterns of distribution of active ingredients and their persistence in plants, seeding rates could impact the effectiveness of different kinds of seed treatments differentially. In the present study, seeds treated at constant per seed treatment rates with chlorantraniliprole, thiamethoxam, or clothianidin were sown at different seeding rates and reductions in population densities of rice water weevil larvae were assessed to investigate possible effects of seeding rate on efficacy of insecticidal seed treatments.

## 2. Experimental

Four experiments were conducted during the 2009, 2010, and 2011 growing seasons at the Louisiana State University Agricultural Center Rice Research Station, Crowley, Acadia Parish, LA, on Crowley silt-loam soils (fine, montmorillonitic, thermic typic albaqualf). All experiments were randomized complete block experiments incorporating factorial combinations of seeding rates and insecticide treatments as described below. For the experiment conducted in 2009, “CL 171”, a widely-grown long-grain rice variety tolerant to the imidazolinone class of herbicides, was used. For the 2010 experiment, “Cheniere”, a widely planted conventional long-grain variety, was used. The first experiment in 2011 utilized “CL 171”, whereas the second experiment utilized “Cocodrie”, another widely grown conventional long-grain variety. Practices for weed, nutrient and water management followed the recommendations of the Louisiana State University AgCenter for drill-seeded rice [24]. Population densities of rice water weevils are consistently high at this experimental site, and no other early- or mid-season pest insects were present in significant numbers at this site over the three years of the study.

Rice in all experiments was drill-seeded into conventionally tilled seedbeds using a grain drill mounted on a tractor. Recommended seeding rates for drill-seeded rice in Louisiana range from 56 to 90 kg·ha^−1^ for conventional (non-hybrid) rice, whereas seeding rates for hybrids in drill-seeded culture can be as low as 23 kg·ha^−1^. Seeding rates used in these experiments were representative of these recommended rates. In 2009 and 2010, seeding rates employed were 34, 68 and 102 kg·ha^−1^ (corresponding to 122, 243 and 365 seeds/m^2^). For the first experiment in 2011, seeding rates were 23, 34 and 68 kg·ha^−1^ (81, 122 and 243 seeds·m^−2^); for the second experiment, in 2011, seeding rates were seeding rates were 34, 68 and 102 kg·seed·ha^−1^. The planting dates for the four experiments were 11 May (2009), 5 May (2010), 9 May (2011, experiment 1), and 16 May (2011, experiment 2).

Insecticide formulations used to treat seeds were Cruiser Maxx™ and Cruiser 5 FS (thiamethoxam 26.4% and 47.6%, respectively; supplied by Syngenta Corporation), Nipsit INSIDE™ (clothianidin 47.5%; supplied by Valent USA) and Dermacor X-100 (chlorantraniliprole 50%; supplied by DuPont Crop Protection). For the 2009 and 2010 experiments and the second experiment in 2011, only thiamethoxam and chlorantraniliprole seed treatments were evaluated. Seed treatment rates in the 2009 experiment were 27 µg AI·seed^−1^ for thiamethoxam and 25 μg AI seed^−1^ for chlorantraniliprole. In 2010, the per seed thiamethoxam rate was 30 µg AI seed^−1^ while the per seed chlorantraniliprole rate was 17 µg·AI·seed^−1^. For the second experiment in 2011, seed treatment rates were 19 μg·AI·seed^−1^ for chlorantraniliprole and 34 μg·AI·seed^−1^ for thiamethoxam. For the first experiment in 2011, thiamethoxam, clothianidin and chlorantraniliprole seed treatments were evaluated at the rates of 32, 17, and 23 μg·AI·seed^−1^, respectively. All rates are within the range of commercial label rates for each insecticide. Seeds were either treated by the manufacturer or treated manually in the lab in small batches. To treat seeds manually, appropriate volumes of each insecticide formulation were mixed with a Brilliant blue dye (Sigma-Aldrich, St. Louis, MO, USA) solution, applied to seeds using a pipettor, and mixed in a rotatory drum to ensure uniform coating of insecticide on seed. Seeds not treated with insecticide but treated with dye alone were used as control (0 µg·AI/seed). In all years, seeds were freshly treated for use in experiments.

In each year, the area in which the experiment was conducted was surrounded by levees on all sides and access to irrigation water was provided by a pipe to a lateral. Each plot measured 5.4 m × 1.8 m with seven rows of rice spaced 17.5 cm apart. Each plot was separated from neighboring plots by at least 1.2 m on all sides. Plots were flushed with well water from a lateral as necessary to establish stands of rice. Permanent flood was established at the 3–4 leaf (early tillering) stage of rice on 1 June (2009), 28 May (2010), 2 June (2011, experiment 1), and 15 June (2011, experiment 2).

For experiments in 2011, plant stands were evaluated when plants reached three- to four-leaf stage to verify the effect of seeding rate on plant stand densities [20]. Plant stand densities were estimated by counting the number of seedlings present in two areas of 0.09 m^2^ (1 foot^2^) per plot. Densities of rice water weevil immatures (larvae and pupae) were determined at one or more time points after flooding by using a soil-root core sampler with diameter of 9.2 cm and a depth of 7.6 cm. Core sampling was done between three and five weeks after flooding. Three core samples were taken from each plot at three sampling dates (18, 28, and 47 days after permanent flood) in 2009. Three core samples were taken from each plot on a single sampling date (20 days post-flood) in 2010. For both 2011 experiments, four core samples were taken from each plot on a single sampling date (26 and 22 days after permanent flood for the first and second experiment, respectively). Core samples were processed by placing them in a sieve bucket (40-mesh screen) and washing soil from roots. Buckets were then placed into basins of salt water, and larvae were counted as they floated to the surface of salt solution and pupal counts were done as they settle in bottom of sieve bucket [25]). Rice was harvested at grain maturity using a mechanical small-plot harvester/thresher and yields were recorded. Four of seven rows of each plot were harvested.

A mean immature density was obtained for each plot by averaging the number of immature weevils (larvae and pupae) found in the three or four soil cores from each plot. The impacts of seeding rate, insecticide treatment, and their interaction on mean larval densities and plant stands were analyzed by analysis of variance (ANOVA) in PROC MIXED using SAS [26]. The data for the 2009 experiment were analyzed by repeated measures ANOVA. To estimate appropriate degrees of freedom, the Kenward-Roger method for adjustment of degrees of freedom was used in the model statement for all experiments. The impact of weevil feeding on grain yield was assessed using ANOVA after adjustment of grain yields from plots to 12% moisture.

## 3. Results

In 2009, densities of rice water weevil larvae and pupae decreased with increasing seeding rates (*F*_2,78_ = 4.2; *p* = 0.02) (Table 1): the lowest immature densities were found in plots with highest seeding rate *i.e.*, 102 kg/ha, whereas densities were intermediate and lowest at the 68 and 34 kg/ha seeding rates, respectively. Insecticide seed treatment significantly reduced population densities of immature *L. oryzophilus* (*F*_2,78_ = 69.8; *p* < 0.0001) (Table 2). Although both seed treatments significantly suppressed insect numbers, suppression in chlorantraniliprole-treated plots was greater than in thiamethoxam treated plots. The interaction between seeding rate and seed treatment was not significant (*F*_4,78_ = 0.3; *p* = 0.9). Reductions in *L. oryzophilus* densities in chlorantraniliprole-treated plots ranged from 79.7% at the 34 kg/ha seeding rate to 90.4% at the 68 kg/ha seeding rate (Table 3). Reductions in *L. oryzophilus* densities in thiamethoxam-treated plots ranged from 49.7% at the 34 kg/ha seeding rate to 59% at the 102 kg/ha seeding rate (Table 3).

**Table 1:** Impact of seeding rate as a main effect on immature weevil densities in small-plot field experiments during 2009–2011 ^a^.

Year	No. Immature Weevils/Core Sample @ Seeding Rate			
	23 kg·ha^−1^	34 kg·ha^−1^	68 kg·ha^−1^	102 kg·ha^−1^
2009	-	14.7 ± 1.5 a	11.5 ± 1.5 b	10.0 ± 1.5 c
2010	-	14.1 ± 1.0 a	11.0 ± 1.0 ab	10.3 ± 1.0 b
2011 Experiment 1	22.7 ± 1.3 a	20.6 ± 1.3 ab	18.4 ± 1.3 b	-
2011 Experiment 2	-	30.0 ± 2.9	28.0 ± 2.9	30.3 ± 2.9

^a^ Densities are least square means; means for each year accompanied by the same letter are not significantly different from each other after Tukey’s adjustment.

**Figure 1:**
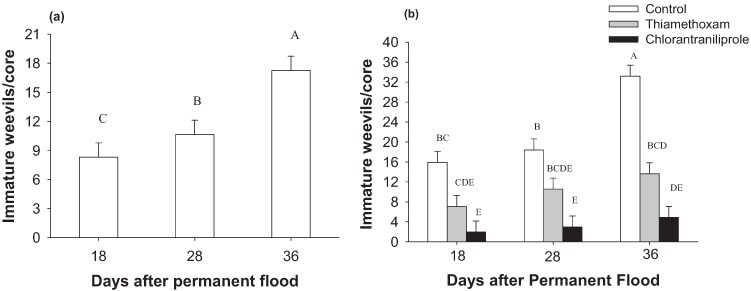
Impact of sampling time on immature weevil densities (**a**) and interaction of sampling time and insecticide treatment on weevil densities (**b**) in the 2009 experiment.

Insect densities were also significantly impacted in 2009 by time of sampling (*F*_2,78_ = 15.9; *p* < 0.0001) (Figure 1a). Densities increased with sampling date: immature densities were lowest at 18 days after permanent flood, intermediate at 28 days, and highest at 36 days after permanent flood. The interaction between seed treatment and time of sampling was significant on insect densities (*F*_4,78_ = 4.4; *p* = 0.003) (Figure 1b). Insect numbers fluctuated more over time on untreated rice than on insecticide- treated rice. Chlorantraniliprole seed treatments significantly reduced insect numbers at all sampling times compared to controls (no seed treatment). Compared to control plots, thiamethoxam seed treatment did not reduce insect numbers significantly at 18 and 28 days after permanent flood (Figure 1b).

**Table 2:** Impact of insecticidal treatment as a main effect on immature weevil densities in small-plot field experiments during 2009–2011 ^a^.

Year	No. Immature Weevils/Core Sample			
	Control	Thiamethoxam	Clothianidin	Chlorantraniliprole
2009	22.5 ± 1.5 a	10.4 ± 1.5 b	-	3.3 ± 1.5 c
2010	17.1 ± 1.0 a	11.6 ± 1.0 b	-	6.8 ± 1.0 c
2011 Experiment 1	26.6 ± 1.3 a	21.3 ± 1.3 b	20.3 ± 1.3 b	10.0 ± 1.3 c
2011 Experiment 2	44.1 ± 2.9 a	36.3 ± 2.9 a	-	8.1 ± 2.9 b

^a^ Densities are least square means; means for each year accompanied by the same letter are not significantly different from each other after Tukey’s adjustment.

**Table 3:** Percent suppression of immature weevils by rice seed treatments under different seeding rates in small-plot experiments during 2009–2011 ^a^.

Year	Seed Treatment	% Suppression at Different Seeding Rates (kg/ha)			
		23	34	68	102
2009	Thiamethoxam	-	49.7 ± 8.5	53.8 ± 8.5	59.0 ± 8.5
	Chlorantraniliprole	-	79.7 ± 8.5	90.4 ± 8.5	87.4 ± 8.5
2010	Thiamethoxam	-	26.6 ± 5.3	27.0 ± 5.3	44.0 ± 5.3
	Chlorantraniliprole	-	47.9 ± 5.3	69.9 ± 5.3	65.4 ± 5.3
2011 Experiment 1	Thiamethoxam	5.8 ± 7.6	9.4 ± 7.6	43.5 ± 7.6	-
	Clothianidin	8.5 ± 7.6	38.7 ± 7.6	22.9 ± 7.6	-
	Chlorantraniliprole	36.2 ± 7.6	42.5 ± 7.6	62.4 ± 7.6	-
2011 Experiment 2	Thiamethoxam	-	23.2 ± 6.6	7.4 ± 6.6	21.1 ± 6.6
	Chlorantraniliprole	-	76.6 ± 6.6	82.6 ± 6.6	85.5 ± 6.6

^a^ % suppression in each year is expression of the decrease in insect densities in treated plots over untreated plots calculated by using (weevil densities in treatment-weevil densities in control)* 100/(weevil densities in control).

Yields in 2009 were significantly impacted both by seeding rate (*F*_2,24_ = 9.6; *p* = 0.0009) (Table 4) and seed treatment (*F*_2,24_ = 38.7; *p* < 0.0001) (Table 5) but not by the interaction of seeding rate and seed treatment (*F*_4,24_ = 0.7; *p* = 0.60) Yields at the 34 kg/ha seeding rate were lower than at 68 and 102 kg/ha. The difference in yields between the latter two seeding rates was not statistically significant. Seed treatment with both thiamethoxam and chlorantraniliprole increased yields. Yields from chlorantraniliprole-treated plots did not differ statistically from yields from thiamethoxam-treated plots.

As in the 2009 experiment, immature weevil densities in the 2010 experiment were significantly impacted both by seeding rate (*F*_2,27_ = 4.1; *p* = 0.03) (Table 1) and insecticide treatment (*F*_2,27_ = 27.0; *p* < 0.0001) (Table 2). Weevil densities were lowest at the 102 kg·ha^−1^ seeding rate and differed significantly from densities at the 34 kg/ha seeding rate. Similar to 2009, both seed treatments significantly reduced insect numbers, with chlorantraniliprole again providing more suppression of immature weevils (60%) than thiamethoxam (32%) (Table 2). The interaction between seeding rate and seed treatment was not significant (*F*_4,27_ = 0.8; *p* = 0.8). For thiamethoxam, reductions in weevil densities compared to controls ranged from 26.6% at the 34 kg/ha seeding rate to 44.0% at the 102 kg/ha seeding rate; for chlorantraniliprole, reductions in weevil densities ranged from 47.9% at 34 kg/ha to 65.4% at 102 kg/ha (Table 3).

**Table 4:** Impact of seeding rate on yields harvested from small plot field experiments during 2009–2011 ^a^.

Year	Yield (kg/ha) at Seeding Rates (kg/ha)			
	23	34	68	102
2009	-	6996 ± 154 ^b^	7689 ± 154 ^a^	7687 ± 154 ^a^
2010	-	8392 ± 228	8407 ± 228	8335 ± 228
2011 Experiment 1	7698 ± 294	7612 ± 294	7332 ± 294	-
2011 Experiment 2		4323 ± 117	4677 ± 117	4485 ± 117

^a^ Yields were adjusted to 12% moisture; yields in each year followed by the same letter are not significantly different from each other after Tukey’s adjustment.

**Table 5:** Impact of seed treatments on yields from small plot field experiments during 2009–2011 ^a^.

Year	Yield (kg/ha) in Different Seed Treatments			
	Control	Thiamethoxam	Chlorantraniliprole	Clothianidin
2009	6568 ± 154 b	7683 ± 154 a	8122 ± 154 a	-
2010	8001 ± 228	8480 ± 228	8653 ± 228	-
2011 Experiment 1	7375 ± 294	7437 ± 294	7791 ± 294	7949 ± 294
2011 Experiment 2	3846 ± 117 c	4577 ± 117 b	5061 ± 117 a	-

^a^ Yields were adjusted to 12% moisture; yields in each year followed by the same letter are not significantly different from each other after Tukey’s adjustment.

Unlike 2009, grain yields in 2010 were not impacted significantly by seeding rate (*F*_2,24_ = 0.03; *p* = 0.97) (Table 4), seed treatment (*F*_2,24_ = 2.4; *p* = 0.1) (Table 5), or the interaction of seeding rate and insecticide treatment (*F*_4,24_ = 1.1; *p* = 0.4).

In 2011, stand densities increased with increasing seeding rate. In the first experiment in 2011, plant densities at 68 kg/ha were significantly higher than at 23 or 34 kg/ha seeding rates. (*F*_2,33_ = 57.6; *p* < 0.0001). In the second experiment in 2011, plant densities at 102 kg/ha seeding rate were significantly higher than plant densities at the 34 and 68 kg/ha seeding rates (*F*_2,24_ = 133.2; *p* < 0.0001). In the second 2011 experiment, plant densities were also higher in insecticide-treated plots than in untreated plots (*F*_2,24_ = 4.3; *p* = 0.03).

**Figure 2:**
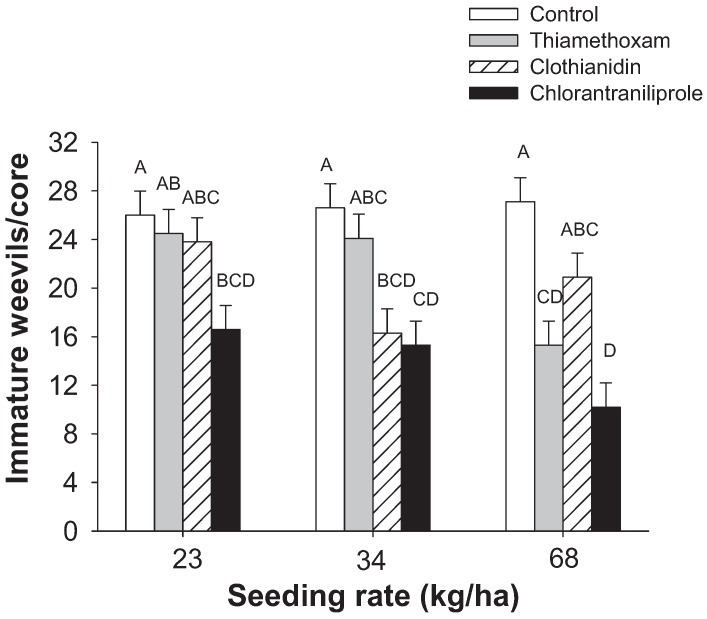
Densities of immature rice water weevils at different seeding rates of rice as impacted by different seed treatments in the first 2011 experiment. Bars accompanied by the same letter represent means that do not differ significantly.

In the first experiment in 2011, weevil densities were significantly impacted by seeding rate (*F*_2,33_ = 5.7; *p* = 0.008) (Table 1) and seed treatment (*F*_3,33_ = 23.8; *p* < 0.0001) (Table 2). Densities were significantly higher at the 23 kg/ha than the 68 kg/ha seeding rate (Table 1). Although all seed treatments reduced weevil densities significantly, weevil suppression was greatest in chlorantraniliprole plots (62%) and suppression by chlorantraniliprole was significantly greater than suppression by clothianidin (24%) and thiamethoxam (20%) (Table 2). Unlike previous years, the interaction between seeding rate and seed treatment was significant (*F*_6,33_ = 3.4; *p* = 0.01) (Figure 2). Seeding rates did not impact weevil numbers significantly in the absence of chemical treatments, but they did impact weevil numbers in insecticide-treated plots. The reduction in weevil densities in thiamethoxam-treated plots was greater at the 68 kg/ha seeding rate than at the 23 kg/ha seeding rate (Figure 2).

Grain yields were not impacted significantly by seeding rate (*F*_2,33_ = 2.1; *p* = 0.1) (Table 4), insecticide treatment (*F*_3,33_ = 1.2; *p* = 0.3) (Table 5), or the interaction of seeding rate and insecticide treatment (*F*_6,33_ = 1.4; *p* = 0.2).

In the second experiment in 2011, weevil densities were not impacted by seeding rate (*F*_2,24_ = 0.2) (Table 1), but were impacted by seed treatment (*F*_2,24_ = 60.2; *p* <0.0001) (Table 2). Suppression of immature weevil densities was greatest in chlorantraniliprole-treated plots (82%) and suppression in these plots was greater than in thiamethoxam-treated plots (18%). Suppression of immature weevils by thiamethoxam treatment was not significant relative to control plots (Table 1). The interaction between seeding rate and seed treatment did not significantly impact weevil densities (*F*_2,24_ = 0.2; *p* = 0.8). The reduction in weevil densities in chlorantraniliprole-treated plots ranged from 76.6% at 34 kg/ha to 85.5% at 102 kg/ha seeding rate (Table 3).

The impact of seeding rate on grain yields was not significant (*F*_2,24_ = 2.4; *p* = 0.1) (Table 4), but seed treatments significantly impacted grain yields (*F*_2,24_ = 29.3; *p* < 0.0001). Both insecticide treatments significantly increased grain yields; yields from chlorantraniliprole-treated plots were highest and were greater than yields from thiamethoxam-treated plots (*p* = 0.01) (Table 5). The interaction of seeding rate and insecticide treatment did not significantly affect yields in this experiment (*F*_4,24_ = 0.6; *p* = 0.67).

## 4. Discussion

Rice in the southern U.S. is frequently seeded at low rates (25 to 60 kg of seed per ha) [23] and treated before planting with neonicotinoid (thiamethoxam, clothianidin) or anthranilic diamide (chlorantraniliprole) seed treatments. It is therefore important to understand the potential interactions among these two practices. Insecticidal seed treatments are thought to affect rice water weevils via systemic movement of active ingredient in the plant and ingestion of active ingredient by the insects as they feed on leaves or roots [10,11,12]. If this is indeed the case, then the effectiveness of seed treatments should depend on per seed treatment rate but not on seeding rate. If, on the other hand, rice water weevils are also affected when insecticide leaches into the soil and comes into contact with larvae as they feed on rice roots, low seeding rates might impact the efficacy of insecticidal seed treatments by reducing the amount of insecticide present in soil. This is because, given a constant per seed treatment rate, the amount of insecticide per unit volume of soil should decrease as seeding rates decrease.

The four experiments reported here were designed to test the hypothesis that low seeding rates compromise the effectiveness of seed treatments (*i.e.*, that reductions in population densities of rice water weevil larvae in insecticide-treated plots are greater at higher seeding rates than at lower seeding rates). In only one of four experiments did the interaction between rice seeding rate and seed treatment significantly affect densities of immature rice water weevils. Furthermore, in the one experiment in which a significant interaction was found, it was only the activity of thiamethoxam that was compromised at low seeding rates. In the remaining experiments, efficacies of seed treatments were statistically independent of seeding rates. Thus, these experiments provided little evidence for the hypothesis that low seeding rates compromise the effectiveness of seed treatments, and strongly indicate that the primary route by which these seed treatments affect weevils is via systemic transport in the plant and ingestion by larval or adult weevils. However, these experiments may not have been sufficiently powerful to detect a weak effect of seeding rate on the effectiveness of seed treatments, and the non-significant trends toward lower suppression of weevils by seed treatments at low seeding rates seen in Table 3 suggest that larger experiments will be needed to fully determine if contact of weevil larvae with insecticide active ingredient in the soil is a secondary route of exposure, or if greater vegetative growth of plants seeded at low rates reduces the effectiveness of seed treatments by diluting insecticide active ingredient.

In contrast to the weak evidence for the effect of seeding rate on insecticide effectiveness, these experiments provided strong evidence for the superior efficacy of chlorantraniliprole seed treatments relative to neonicotinoid seed treatments. The superior efficacy of chlorantraniliprole relative to neonicotionids against the rice water weevil has been previously reported [27,28,29,30]. Across all four experiments in the current study, chlorantraniliprole seed treatment reduced weevil densities by 72% relative to untreated controls, whereas thiamethoxam seed treatments reduced weevil densities by 31%. Chlorantraniliprole seed treatments were more effective despite the fact that per seed treatment rates for chlorantraniliprole (17 to 25 µg·AI·seed^−1^) were always lower than the per seed treatment rates for thiamethoxam (27 to 34 µg·AI·seed^−1^) and were similar to the rate used for clothianidin in the 2011 experiment. This superior efficacy of chlorantraniliprole is likely attributable to a combination of greater inherent toxicity toward *L. oryzophilus* and differences in the within-plant distribution and persistence of chlorantraniliprole relative to the neonicotionoid active ingredients. Neonicotinoids are highly soluble in water and have higher acropetal mobility (basal to apical regions) than chlorantraniliprole in rice plants [10]. As a result of these differences in the chemical properties of the active ingredients, neonicotinoid concentrations tend to be higher in above-ground portions of plants than in roots (where rice water weevil larvae feed), whereas the converse is true of chlorantraniliprole. In addition, analysis of residues in plants treated as seeds at different rates of active ingredient revealed greater persistence of active ingredient in plants treated with chlorantraniliprole than in plants treated with thiamethoxam [30].

Data from these experiments also confirm the effect of seeding rate on population densities of rice water weevil larvae reported earlier by Stout *et al.* [22]. In this previous report, low rice seeding rates, which resulted in low plant densities, were found to be associated with modestly higher levels of infestation by, and yield losses from, the rice water weevil [22]. The magnitude of increase in larval population densities at the lowest seeding rate relative to the highest seeding rate ranged from 24% (2011, experiment 1) to 47% (2009). Yields, however, were affected by seeding rate in only one of the four experiments.

Currently, the label for chlorantraniliprole seed treatment in rice (Dermacor X-100) specifies that seed treatment rates be adjusted for seeding rate to maintain a constant per area rate of insecticide application (*i.e.*, higher seed treatment rates are required at low seeding rates). This provision, coupled with the more robust activity of this insecticide toward the rice water weevil, suggests there are few concerns associated with using Dermacor X-100 in rice seeded at low rates. The results of this study, however, suggest that caution should be exercised when using the less effective neonicotinoid seed treatments in rice seeded at low rates. Although the interaction of seeding rate and insecticidal seed treatment significantly affected population densities of rice water weevils in only one of the four experiments reported here, suppression of weevil populations in neonicotinoid-treated plots seeded at low rates was overall quite poor, averaging only 27% over four experiments. Furthermore, the labels for neonicotinoid seed treatments specify the same seed treatment rates in terms of the amount applied per acre be used regardless of rice seeding rate, and, as a result, per area rates of insecticide decrease with decreasing seeding rates. Not adjusting neonicotinoid seed treatment rates to account for low seeding rates may compound the inherent inferiority of these products against the rice water weevil, and rice seeded at low rates and treated with a neonicotinoid may be at risk for more damaging infestations of rice water weevils than rice seeded at higher rates and treated with the more effective chlorantraniliprole.

## 5. Conclusions

Suppression of larvae by neonicotinoid seed treatments in plots seeded at low rates was sometimes poor, and the results of this study suggest that caution must be exercised by growers when using the less effective neonicotioids at low seeding rates. The efficacy of chlorantraniliprole seed treatment is greater than that of the neonicotionids, and the activity of chlorantraniliprole is not affected by low seeding rate (particularly since the label for this insecticide specifies that per seed rates should be increased when seeding at low rates). Further experiments will be needed to investigate whether contact of weevil larvae with insecticide active ingredient in the soil is a secondary route of exposure, or whether greater vegetative growth of plants seeded at low rates dilutes insecticide active ingredient in plants to a greater degree, thereby reducing the effectiveness of seed treatments.
